# Regional variations and deprivation are linked to poorer access to laparoscopic and robotic colorectal surgery: a national study in England

**DOI:** 10.1007/s10151-023-02874-3

**Published:** 2023-12-11

**Authors:** A. J. Morton, A. Simpson, D. J. Humes

**Affiliations:** 1 Department of Colorectal Surgery, Nottingham University Hospitals NHS Trust, Nottingham, UK; 2 NIHR Nottingham BRC, University of Nottingham and Nottingham University Hospitals NHS Trust, Nottingham, UK

**Keywords:** Laparoscopy, Robotic surgical procedures, Minimally invasive surgical procedures, Colorectal surgery, Health inequalities

## Abstract

**Background:**

Laparoscopic and now robotic colorectal surgery has rapidly increased in prevalence; however, little is known about how uptake varies by region and sociodemographics. The aim of this study was to quantify the uptake of minimally invasive colorectal surgery (MIS) over time and variations by region, sociodemographics and ethnicity.

**Methods:**

Retrospective analysis of routinely collected healthcare data (Clinical Practice Research Datalink linked to Hospital Episode Statistics) for all adults having elective colorectal resectional surgery in England from 1 January 2006 to 31 March 2020. Sociodemographics between modalities were compared and the association between sociodemographic factors, region and year on MIS was compared in multivariate logistic regression analysis.

**Results:**

A total of 93,735 patients were included: 52,098 open, 40,622 laparoscopic and 1015 robotic cases. Laparoscopic surgery surpassed open in 2015 but has plateaued; robotic surgery has rapidly increased since 2017, representing 3.2% of cases in 2019. Absolute differences up to 20% in MIS exist between regions, OR 1.77 (95% CI 1.68–1.86) in South Central and OR 0.75 (95% CI 0.72–0.79) in the North West compared to the largest region (West Midlands). MIS was less common in the most compared to least deprived (14.6% of MIS in the most deprived, 24.8% in the least, OR 0.85 95% CI 0.81–0.89), with a greater difference in robotic surgery (13.4% vs 30.5% respectively). Female gender, younger age, less comorbidity, Asian or ‘Other/Mixed’ ethnicity and cancer indication were all associated with increased MIS.

**Conclusions:**

MIS has increased over time, with significant regional and socioeconomic variations. With rapid increases in robotic surgery, national strategies for procurement, implementation, equitable distribution and training must be created to avoid worsening health inequalities.

**Supplementary Information:**

The online version contains supplementary material available at 10.1007/s10151-023-02874-3.

## Introduction

Laparoscopic colorectal surgery became the standard of care during the first decade of this century [1], with evidence of equivalent oncological and long-term outcomes to open surgery, a quicker postoperative recovery and reduced length of stay (LOS) [2–7]. The increased equipment costs and prolonged operating time may have presented a barrier to uptake [8], but savings related to quicker recovery, reduced LOS and reduced operating time offset these [9–12]. In England, a national laparoscopic training programme was created (LAPCO) to provide standardised training in laparoscopic colorectal surgery to mitigate any impact of a learning-curve effect on outcomes [13]. Robotically assisted colorectal surgery has equivalent oncological outcomes and possible improvements in LOS and conversion rates when compared to laparoscopic surgery [14–18]. Uptake of robotic colorectal surgery is increasing despite high set-up costs (approximately £1.35 million for a robotic platform [19]).

A large, population-based study in the USA showed significant increases in minimally invasive colorectal surgery (MIS) and robotic surgery between 2011 and 2018 [20]. The authors suggested that the reasons for this were likely multifactorial but may include more minimally invasive trained surgeons entering the workforce, increased training of established surgeons or drivers to take advantage of better outcomes in terms of quality of life and LOS. A study using Hospital Episode Statistics (HES) in England showed increasing robotic gynaecological surgery from 2006 to 2018, with regional variations favouring large urban centres [21]. Uptake of robotics in colorectal surgery is linked to uptake in other specialties in that unit, showing institutional factors are important in driving this growth [22]. However, despite increasing uptake, only approximately 25% of National Health Service (NHS) Trusts had a robotic platform—mainly large, urban hospitals with no access for some rural areas [19]. Despite the advantages of minimally invasive approaches being described, there has been little research describing the adoption of the techniques and how these may vary by region and patient characteristics.

Patients from ethnic minority groups and lower socioeconomic status are less likely to receive MIS for renal cancer in the USA [23]; however, there is little national data from England describing the uptake and regional, demographic and ethnic variations of MIS in colorectal surgery. This has important implications as the uptake of robotic surgery is increasing; without careful planning, the high set-up costs may widen pre-existing health inequalities.

This study aims to use a large, population-based dataset to describe the uptake of MIS in colorectal surgery over time, and variations by region, ethnicity, socioeconomic status and demographics.

## Methods

The study was approved by the Independent Scientific Advisory Committee (ID 21_000572) and follows STROBE guidelines.

### Data source

This is a population-based, retrospective observational study using routinely collected healthcare data. The Clinical Practice Research Datalink (CPRD) Aurum database contains anonymised primary care data for use in clinical research. Over 13 million currently contributing patients from 1489 practices in England (approximately 20% of the population) [24] are included and it is representative of the national population [25]. Information is linked to HES (a record of each episode of inpatient secondary care delivered in, or funded by, NHS healthcare providers in England), region and deprivation data. HES provides International Classification of Disease (ICD-10) diagnosis codes, Office of Population, Censuses and Surveys (OPCS-4) codes for surgical procedures, admission and discharge dates.

### Patient cohort

**Fig. 1:**
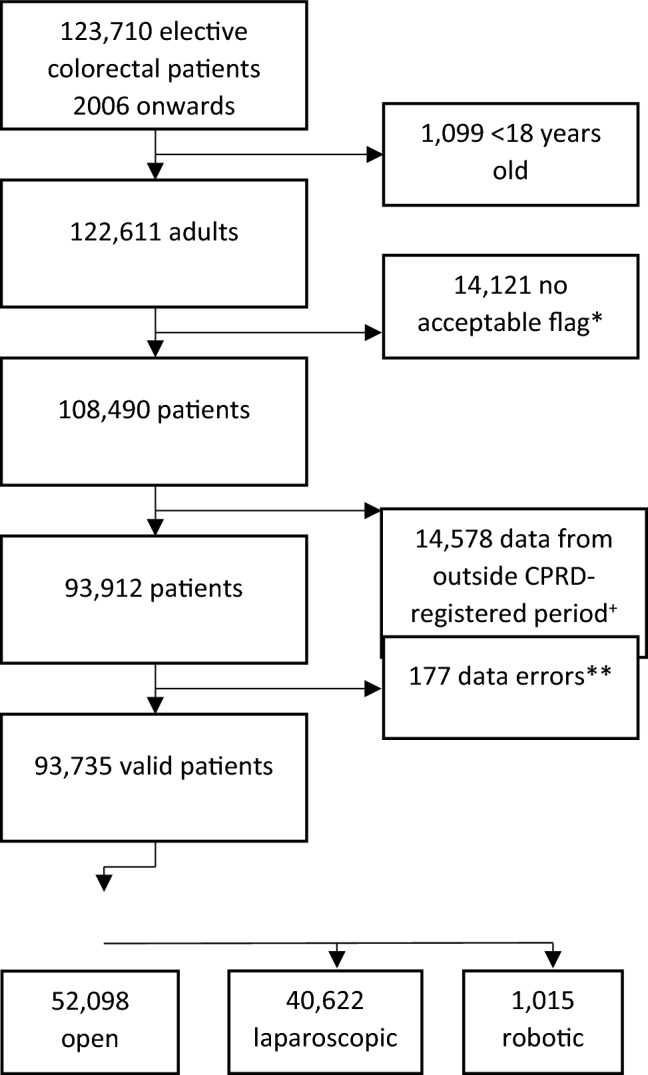
Flow diagram of patients included in study. *CPRD flag for acceptable quality of data,  ^+^data includes period outside practice registration with CPRD,  **no event date, episode start date after discharge, death prior to episode start, age > 110

The cohort included all patients with an OPCS code for elective colectomy (Appendix 1) within HES linked CPRD AURUM data, recorded between 1 January 2006 and 31 March 2020. Exclusion criteria included age < 18 years, emergency or uncoded admission in HES, uncoded operation date, data conflicts (e.g. death prior to operation date, data recorded outside of primary care practice CPRD registration date, age > 110 years) or not meeting CPRD quality measures (recommended by CPRD for research-quality data: “acceptable” flag for individual patient data and “up-to-standard” dates for practice-level data recording, Fig. 1).

### Outcomes

The primary outcome was to determine uptake of MIS over time and how this varied by sociodemographic factors.

### Exposures

Operations were defined from HES using OPCS-4 codes for colectomy and supplementary codes for identification of MIS (Appendix 1). Operations that started with a minimally invasive approach were classed as MIS. Year of operation was recorded from date of colectomy. Region of GP practice was taken from CPRD and Index of Multiple Deprivation (IMD) quintiles were linked. Indication for operation was obtained from ICD-10 discharge codes associated with the primary admission for surgery (Appendix 1). Where patients had multiple ICD-10 codes, only one indication was given with the following priority: cancer, inflammatory bowel disease, diverticular disease, other. Age was divided into four categorical groups (18–39, 40–59, 60–79, 80+). Ethnicity was categorised into ‘White’, ‘Asian’, ‘Black’ and ‘Other/Mixed’ (including unknown ethnicity). Comorbidity was classified according to Charlson index prior to admission, categorised as 0, 1 or ≥ 2. ‘Left-sided’ operations were considered those involving removal of the descending or sigmoid colon, or rectum.

### Statistical analysis

Demographic information was compared with chi-squared between surgical modalities. For the outcome MIS, the impact of various exposures (gender, age, Charlson score, ethnicity, deprivation measured with IMD, indication for surgery, year of operation and region) were assessed using univariate logistic regression. Exposures significantly associated with MIS were included in the multivariate model, with interactions assessed using likelihood ratio tests. Analysis was undertaken using Stata SE version 17 (Statacorp, Texas, USA).

## Results

### Demographics

A total of 93,735 patients were suitable for analysis (Fig. 1), including 40,622 laparoscopic and 1015 robotic cases; 51.1% were male, 48.9% female. Median age was 66.9 years (IQR 54.8–75.8): 67.4 years (IQR 56.2–76.3 years) in open surgery, 66.1 years (IQR 52.3–75.2 years) in laparoscopic and 65.2 years (IQR 55.9–73.2 years) in robotic (*p* < 0.001).

Significant comorbidity (Charlson 2+) was greater in open surgery (75.1%) and robotic surgery (81.8%) than laparoscopic (73.3%), *p* < 0.001. In terms of ethnicity, 92.1% of the cohort were of White ethnicity, 2.7% Asian ethnicity, 2.0% Black ethnicity and 3.2% ‘Other/Mixed’ (of which 1595 (1.7%) were unknown). The greatest proportion of MIS (90.8%) was for patients of White ethnicity, 3.1% for Asian ethnicity, 2.1% for Black ethnicity and 4.0% for ‘Other/Mixed’ ethnicities. For robotics, 86.8% of cases were for patients of White ethnicity, 3.4% for Asian ethnicity, 1.7% for Black ethnicity and 8.2% for ‘Other/Mixed’ (Table 1).

**Table 1:** Demographics

Variable	Open (%) *n* = 52,098	Laparoscopic (%) *n* = 40,622	Robot (%) *n* = 1015	Any MIS (%) *n* = 41,637
Sex^a^				
Male	27,198 (52.21)	20,119 (49.53)	603 (59.41)	20,722 (49.77)
Female	24,900 (47.79)	20,503 (50.47)	412 (40.59)	20,915 (50.23)
Age (years)^a^				
18–39	4090 (7.85)	5391 (13.27)	72 (7.09)	5463 (13.12)
40–59	12,397 (23.80)	9290 (22.87)	286 (28.18)	9576 (23.00)
60–79	27,622 (53.02)	20,331 (50.05)	573 (56.45)	20,904 (50.21)
80+	7989 (15.33)	5610 (13.81)	84 (8.28)	5694 (13.68)
Charlson^a^				
0	8987 (17.25)	7854 (19.33)	109 (10.74)	7963 (19.12)
1	4003 (7.68)	2988 (7.36)	76 (7.49)	3064 (7.36)
2+	39,108 (75.07)	29,780 (73.31)	830 (81.77)	30,610 (73.52)
Indication^a^				
Cancer	33,489 (64.28)	28,562 (70.31)	887 (87.39)	29,449 (70.73)
IBD	4400 (8.45)	3689 (9.08)	15 (1.48)	3704 (8.90)
Diverticular disease	5045 (9.68)	2672 (6.58)	45 (4.43)	2717 (6.53)
Other	9164 (17.59)	5699 (14.03)	68 (6.70)	5767 (13.85)
Operation site^a^				
Right and transverse	15,833 (30.39)	14,847 (36.55)	156 (15.37)	15,003 (30.39)
Left-sided*	26,365 (50.61)	18,676 (45.98)	676 (66.60)	19,352 (46.48)
AP resection	3471 (6.66)	2078 (5.12)	123 (12.12)	2201 (5.29)
Other colectomy	6429 (12.34)	5021 (12.36)	60 (5.91)	5081 (12.20)
Ethnicity^a^				
White (*n* = 86,306, 92.07%)	48,498 (93.09)	36,927 (90.90)	881 (86.80)	37,808 (90.80)
Asian (*n* = 2530, 2.70%)	1228 (2.36)	1268 (3.12)	34 (3.35)	1302 (3.13)
Black (*n* = 1866, 1.99%)	1007 (1.93)	842 (2.07)	17 (1.67)	859 (2.06)
Other/Mixed (*n* = 3033, 3.24%)	1365 (2.62)	1585 (3.90)	83 (8.18)	1668 (4.01)
IMD**^a^				
1	11,526 (22.15)	10,003 (24.65)	309 (30.47)	10,312 (24.80)
2	11,206 (21.54)	9011 (22.21)	209 (20.61)	9220 (22.17)
3	10,686 (20.54)	8226 (20.27)	193 (19.03)	8419 (20.24)
4	9866 (18.96)	7402 (18.24)	167 (16.47)	7569 (18.20)
5	8750 (16.82)	5932 (14.62)	136 (13.41)	6068 (14.57)

*Excluding AP resection

**Index of multiple deprivation (IMD), least to most deprived. In total, 113 missing values were not included

^a^Chi-squared between open, laparoscopic and robotic groups all *p* < 0.001

Nearly all (99.9%) of the cohort had deprivation data (113 missing). Affluent patients were more likely to get MIS, with the least deprived IMD quintile representing 24.8% of MIS compared to 14.6% in the most deprived. This difference was greater in robotic surgery, with 30.5% of robotic cases in the least deprived quintile compared to 13.4% in the most deprived. This was driven largely by a much higher proportion of robotic surgery in the most affluent quintile than any other group.

Indication for surgery was colorectal cancer in 67.1%, inflammatory bowel disease (IBD) in 8.7%, diverticular disease in 8.3% and other in 15.9%. Cancer indication was more common in MIS: 70.7% in laparoscopic and 87.4% in robotic compared to 64.3% in open, *p* < 0.001. Laparoscopic surgery was more commonly performed for IBD (9.1%) than open surgery (8.45%), with few robotic cases performed for IBD (1.5%), *p* < 0.001. Open surgery was more likely to be left-sided (57.3%) than laparoscopic (51.1%), but robotic surgery was far more likely to be left-sided (78.7%, *p* < 0.001).

### Uptake over time and by region

**Fig. 2:**
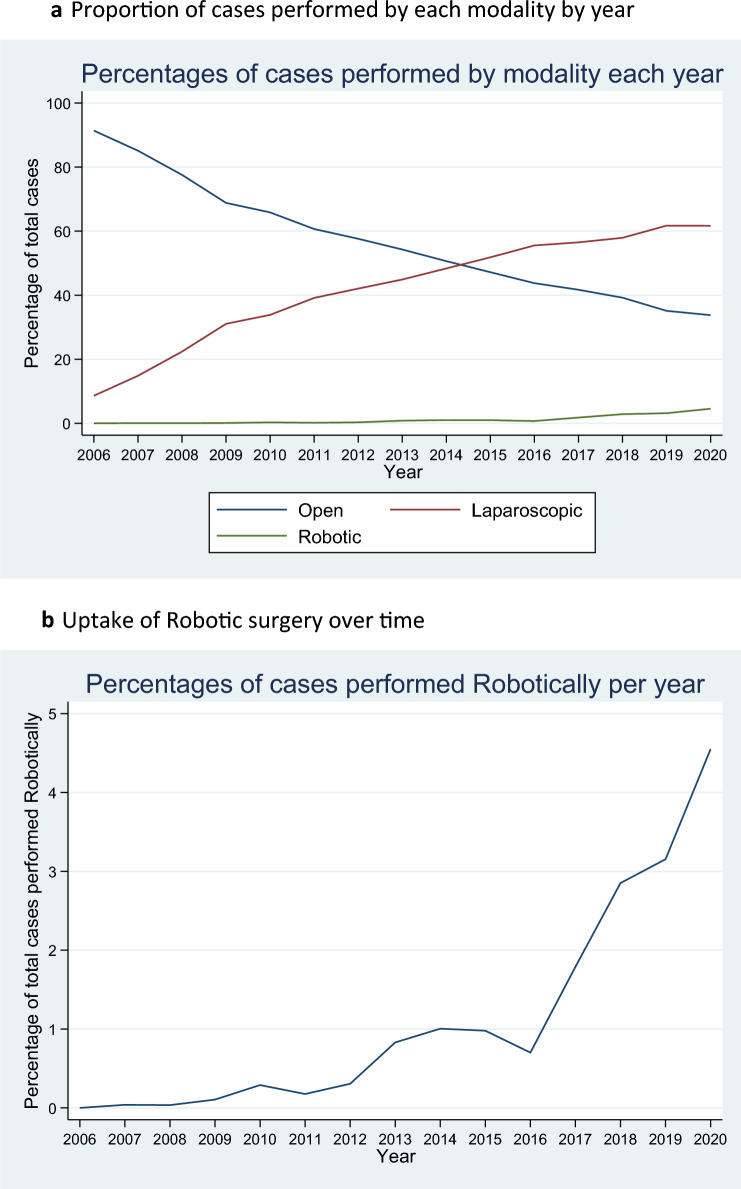
**a** Proportion of cases performed by each modality by year. **b** Uptake of robotic surgery over time

Over time, there has been a significant transition from open surgery (91.4% of cases in 2006) to laparoscopic surgery (61.7% of cases in 2019). Laparoscopic overtook open surgery as the most common modality in 2015 but has started to plateau (Fig. 2a). Robotic surgery is increasing exponentially, with rapid increase since 2017, representing 3.2% of colectomies in 2019 (Fig. 2b).

**Fig. 3:**
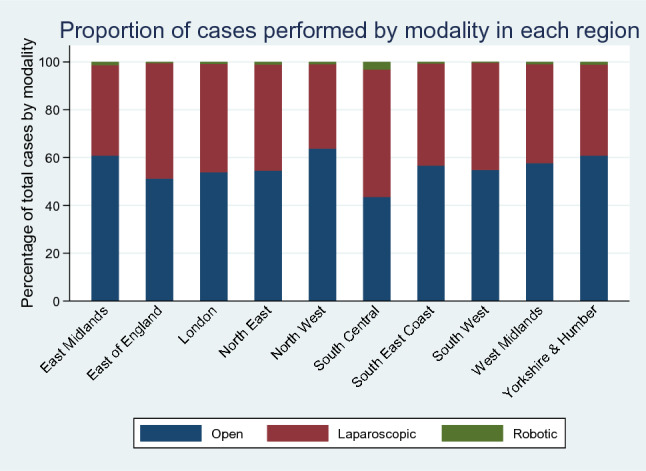
Proportion of cases performed by modality in each region since 2006

Large variations in MIS exist between regions within England, from 36.3% of cases in the North West to 56.4% in South Central (Fig. 3). These significant differences between regions exist for both laparoscopic and robotic surgery (*χ*^2^ *p* < 0.001). Uptake of MIS varies over time by region: regions with a lower proportion of laparoscopic surgery have remained persistently lower over time; similar trends are starting to be observed for robotic surgery (Supplementary Fig. 1). In 2019, this ranged from 48.6% (East Midlands) to 69.3% (North East) for laparoscopic surgery, and 0.6% (South West) to 7.3% (South Central) for robotic cases.

### Logistic regression model for MIS

Gender, age, Charlson score, ethnicity, IMD, surgical indication, year and region were all significantly associated with receiving MIS in univariate analysis and carried forward into a multivariate model (Table 2).

**Table 2:** Logistic regression for factors impacting MIS

Exposure	Open (%) *n* = 52,098	MIS (%) *n* = 41,637	Univariate OR (95% CI)	Multivariate OR (95% CI)
Gender				
Male	27,198 (52.21)	20,722 (49.77)	1.00^a^	1.00^a^
Female	24,900 (47.79)	20,915 (50.23)	1.10 (1.07–1.13)	1.15 (1.12–1.19)
Age (per year)			0.99 (0.99–0.99)	0.99 (0.99–0.99)
Charlson score				
0	8987 (17.25)	7963 (19.12)	1.00^a^	1.00^a^
1	4003 (7.68)	3064 (7.36)	0.86 (0.82–0.91)	0.80 (0.75–0.85)
2+	39,108 (75.07)	30,610 (73.52)	0.88 (0.85–0.91)	0.73 (0.70–0.76)
Ethnicity				
White	48,498 (93.09)	37,808 (90.80)	1.00^a^	1.00^a^
Asian	1228 (2.36)	1302 (3.13)	1.36 (1.26–1.47)	1.16 (1.06–1.26)
Black	1007 (1.93)	859 (2.06)	1.09 (1.00–1.20)	0.97 (0.87–1.07)
Other/Mixed	1365 (2.62)	1668 (4.01)	1.57 (1.46–1.69)	1.10 (1.01–1.19)
IMD*				
1	11,526 (22.15)	10,312 (24.80)	1.00^a^	1.00^a^
2	11,206 (21.54)	9220 (22.17)	0.92 (0.89–0.96)	0.97 (0.93–1.01)
3	10,686 (20.54)	8419 (20.24)	0.88 (0.85–0.92)	0.94 (0.90–0.98)
4	9866 (18.96)	7569 (18.20)	0.86 (0.82–0.89)	0.90 (0.86–0.94)
5	8750 (16.82)	6068 (14.57)	0.78 (0.74–0.81)	0.85 (0.81–0.89)
Indication				
Cancer	33,489 (64.28)	29,449 (70.73)	1.00^a^	1.00^a^
IBD	4400 (8.45)	3704 (8.90)	0.96 (0.91–1.00)	0.61 (0.57–0.64)
Diverticular disease	5045 (9.68)	2717 (6.53)	0.61 (0.58–0.64)	0.49 (0.46–0.51)
Other	9164 (17.59)	5767 (13.85)	0.72 (0.69–0.74)	0.47 (0.45–0.49)
Increasing year			1.19 (1.18–1.19)	1.19 (1.19–1.20)
Region				
West Midlands	9458 (18.2)	6929 (16.6)	1.00^a^	1.00^a^
North East	1993 (3.8)	1666 (4.0)	1.14 (1.06–1.23)	1.23 (1.14–1.33)
North West	9902 (19.0)	5630 (13.5)	0.78 (0.74–0.81)	0.75 (0.72–0.79)
Yorkshire & Humber	2182 (4.2)	1404 (3.4)	0.88 (0.82–0.95)	0.87 (0.80–0.94)
East Midlands	1304 (2.5)	838 (2.0)	0.88 (0.80–0.96)	0.83 (0.75–0.91)
East of England	2214 (4.3)	2111 (5.1)	1.30 (1.22–1.39)	1.34 (1.25–1.44)
South West	7086 (13.6)	5840 (14.0)	1.12 (1.07–1.18)	1.16 (1.10–1.22)
South Central	5240 (10.1)	6786 (16.3)	1.77 (1.69–1.85)	1.77 (1.68–1.86)
London	7692 (14.8)	6585 (15.8)	1.17 (1.12–1.22)	1.11 (1.05–1.16)
South East Coast	5018 (9.6)	3843 (9.2)	1.05 (0.99–1.10)	1.04 (0.98–1.10)

All categories *p* < 0.001

*Index of multiple deprivation, ordered by increasing deprivation

^a^Reference groups presented with OR of 1

Following multivariate analysis female gender was associated with a 15% higher odds of MIS (OR 1.15 95% CI 1.12–1.19) compared to male gender. MIS decreased with each year of increasing age (OR 0.99, 95% CI 0.99–0.99) and with increasing comorbidity (OR 0.73, 95% CI 0.70–0.76 in Charlson 2+ compared to Charlson 0). Compared to White ethnicity, MIS was higher for patients of Asian ethnicity (OR 1.16, 95% CI 1.06–1.26) and ‘Other/Mixed’ (OR 1.09, 95% CI 1.01–1.19), with no difference for those of Black ethnicity (OR 0.97, 95% CI 0.87–1.07). MIS decreased stepwise with increasing IMD quintile, OR 0.85 (95% CI 0.81–0.89) for the most compared to least deprived. There was significantly less MIS for benign compared to malignant indication (IBD OR 0.61, 95% CI 0.57–0.64; diverticular disease OR 0.49, 95% CI 0.46–0.51).

There is an approximate 20% increased odds of a patient receiving MIS each year since 2006 (OR 1.19, 95% CI 1.19–1.20). Compared to the West Midlands, there were significant differences in MIS between regions. The highest likelihood of MIS was in South Central (OR 1.77, 95% CI 1.68–1.86) and lowest in the North West (OR 0.75, 95% CI 0.72–0.79) over the period of the study.

## Discussion

### Key findings

Uptake of minimally invasive colorectal surgery has increased rapidly since 2006, with similar rapid growth now seen in robotics from 2017. However, there are significant variations across different regions and different socioeconomic, ethnic and demographic groups.

Differences in MIS between regions have persisted over time. In 2019, a large absolute difference of approximately 20% exists between individual regions (69.3% MIS in the North East vs 48.6% in the East Midlands), disadvantaging patients in these regions from the benefits of MIS. Robotically assisted surgery shows larger discrepancies, with some regions doing very little (from 0.6% in South West to 7.3% in South Central in 2019). The regional inequalities in robotic surgery are likely to be worsened by the high set-up costs, as larger centres will have more funding and specialties to utilise the equipment efficiently, leaving more rural areas underserved [19, 21].

Women, younger patients, cancer indication, Asian or mixed/other ethnicity and more affluent socioeconomic status are all associated with higher usage of MIS. More than double the number of robotic cases were done for the least deprived IMD quintile compared to the most deprived. The much higher proportion of robotic surgery for the most affluent may represent either this group seeking out this modality or more robotic centres in affluent areas.

### Strengths and limitations

This is the first study using national data to assess variations in uptake of colorectal MIS in England. In particular, variations in access for different demographic, ethnic and socioeconomic groups have not previously been assessed. The large numbers of patients included give power to analyse small variations in uptake that may not be seen in smaller datasets. The accuracy of the data has been validated; 93% of patients recorded having robotic surgery in the National Bowel Cancer Audit (NBOCA) also had robotics codes in HES [26]. The regional variation and uptake over time in CPRD is in keeping with previously published literature from Freedom of Information (FOI) requests to trusts [19] and NBOCA [27].

A limitation could include concerns around inaccurate coding for population-based data. However, outcomes from this dataset match those recorded using different methods, suggesting it is accurate [19, 27]. HES also does not provide information on operations performed in the private sector (when not funded by the NHS) which may under-represent the volume of MIS/robotics undertaken within London, the South East and East of England (where private sector activity is the greatest) [27, 28]. However, data compiled by the Nuffield Trust using HES and Private Healthcare Data suggests that only 3.4% of gastrointestinal therapeutic procedures are provided in the private sector, meaning this is likely to account for few patients [28].

### Results in context of other work

These findings fit the national picture of a large increase in laparoscopic surgery over the last decade, with recent increases in robotic surgery [19, 20, 27]. HES-linked data in England has been used to show an increase in robotic surgery for urological [29–31] and gynaecological [21] surgery over the last decade. Uptake of robotics in colorectal surgery has been shown to be linked to uptake in these specialties, likely tied to institutional access to a robotic platform [22]. Some high-quality studies have been done quantifying uptake and variations in robotic colorectal surgery using population-based data in the USA [20] and national FOI requests in England [19], but no national, population-based work has been done in England using CPRD/HES to assess uptake in colorectal surgery. The regional variations of MIS are in keeping with these studies, showing fewer robotic centres in the South West, North West and East of England [19, 21, 27].

A study using HES data for MIS in gynaecological surgery showed significant regional variations and lower rates in the lowest socioeconomic groups [32]. In high MIS centres, rates were similar between socioeconomic groups showing this is likely related to institutions serving poorer areas. The study reported decreased rates of MIS for Black patients compared to White and Asian patients which has not been demonstrated here [32]. The ethnic, age and socioeconomic differences found here may be explained by the fact that MIS uptake has been driven by large centres based in cities, representing younger and more ethnically diverse urban populations compared to the national population [26].

MIS was introduced in the context of cancer. IBD/diverticular disease cases can often be complex as a result of previous inflammation, so it is no surprise that MIS, especially robotics, is associated with cancer indication. The fact that women are more likely to receive MIS is harder to explain; other factors not assessed here, such as body mass index (BMI) or cancer stage, may account for this.

### Significance and implications

This study demonstrates significant regional, socioeconomic and demographic variations for MIS and robotic surgery, a priority for further work from NBOCA [27]. Robotics offers the next step-change in MIS, but the data presented here shows that, despite national training programmes, it has taken 15 years for laparoscopic colorectal surgery to plateau. Significant regional and sociodemographic inequalities persist and without a national strategy a prolonged and unequal uptake will occur for robotic surgery. These variations mean any benefits are not passed down to the patients with most need (highest colorectal cancer mortality in the most deprived) [33, 34].

Studies demonstrate higher volume centres have lower readmission rates after MIS [27, 31], with decreased costs [35]. NHS Scotland has invested £20 million to acquire 10 surgical robots nationally [36] and the Welsh Government has provided £4.2 million in funding (alongside £13.35 million from health boards) to create an ‘All-Wales Robotic Assisted Surgery Network’ [37]. This centralised procurement may allow more equal distribution than the current system in England of local trust procurement, favouring trusts with more capital, which is also heavily influenced by political and contractual considerations, as well as individual members of staff within departments driving uptake of robotics. This could increase equity in access and outcomes, but also focusses robotic surgery (and rectal cancer) on high-volume centres, which may reduce costs but worsen inequalities for rural patients unless accounted for in pathways.

The roll-out of laparoscopic colorectal surgery was associated with short-term increased morbidity and mortality due to a learning-curve effect [38]. A national laparoscopic training scheme was created between 2006 and 2013 in England (LAPCO), where consultant surgeons received one-to-one training at designated national centres, with competency assessment. This training programme achieved significant improvements in laparoscopic cases numbers, mortality, conversion rates and re-intervention rates; however, it has taken over a decade to achieve a plateau in the use of laparoscopic surgery and disparities still exist [13]. With a learning curve for robotic surgery [39], formalised training programmes are important to minimise short-term impact on morbidity and mortality. Multiple structured training programmes are offered by industry, aiming to minimise harm during this ‘learning curve’ [40], but these vary greatly in progression criteria, assessment and supervision [41]. As robotic surgery becomes more common, increased demand on training programmes and a need for consistent outcomes mean that a national strategy for robotic training should be considered, ensuring that outcomes are consistent with trainers in each region.

## Conclusions

Minimally invasive colorectal surgery has increased over the last 15 years and started to plateau, with signs of a rapid increase in robotic surgery in the last few years. Uptake varies greatly by region and demographics, with deprived patients much less likely to have MIS. Ensuring laparoscopic rates are equitable between groups and regions is important as robotic surgery becomes established. Variations risks worsening health inequalities that exist for already disadvantaged groups as robotic surgery increases in prevalence. These groups require attention during health-service planning, as inequalities are likely to widen without a national strategy. A national strategy for robotic implementation and training is essential to ensure equitable care and good outcomes from this novel technology.

## Supplementary Information

Below is the link to the electronic supplementary material.

Supplementary file1 (DOCX 14 KB)

Supplementary file1 (DOCX 36 KB)

## Data Availability

Clinical Practice Research Datalink (CPRD), linked to Hospital Episode Statistics. The data that support the findings of this study are available from CPRD but restrictions apply to the availability of these data, which were used under licence for the current study, and so are not publicly available. Data are however available from the authors upon reasonable request and with permission of CPRD.
