# Supplementary material for: Regional variations and deprivation are linked to poorer access to laparoscopic and robotic colorectal surgery: a national study in England
Source: Tech Coloproctol. 2023 Dec 11;28(1):9. doi: 10.1007/s10151-023-02874-3 (PMC10713759; doi:10.1007/s10151-023-02874-3)
Supplement: Supplementary file 1 — Supplementary file1 (DOCX 14 KB) [file 10151_2023_2874_MOESM1_ESM.docx]

**Appendix 1: Medical codes**

**OPCS Procedure codes:**

H041, H042, H043, H048, H049, H051, H052, H053, H058, H059, H061, H062, H063, H064, H068, H069, H071, H072, H073, H074, H078, H079, H081, H082, H083, H084, H085, H088, H089, H091, H092, H093, H094, H095, H098, H099, H101, H102, H103, H104, H105, H108, H109, H111, H112, H113, H114, H115, H118, H119, H291, H292, H293, H294, H298, H299, H331, H332, H333, H334, H335, H336, H337, H338, H339, H341, H411, H414, H471, H478, H479, H661, H662, H668, H669

**Minimally Invasive Codes**

Y751, Y752, Y753 (robotic), Y754, Y758, Y759

**Colorectal Cancer ICD-10 codes**

C18, C180, C181, C182, C183, C184, C185, C186, C187, C188, C189, C19, C20

**Inflammatory bowel disease codes**

K50, K500, K501, K508, K509, K51, K510, K512, K513, K514, K515, K518, K519, K520, K521, K522, K523, K528, K529

**Diverticular disease codes**

K57, K570, K571, K572, K573, K574, K575, K578, K579
