# Supplementary material for: Regional variations and deprivation are linked to poorer access to laparoscopic and robotic colorectal surgery: a national study in England
Source: Tech Coloproctol. 2023 Dec 11;28(1):9. doi: 10.1007/s10151-023-02874-3 (PMC10713759; doi:10.1007/s10151-023-02874-3)
Supplement: Supplementary file 2 — Supplementary file1 (DOCX 36 KB) [file 10151_2023_2874_MOESM2_ESM.docx]

**Supplementary Fig. 1** Robotic and Laparoscopic Uptake by region over year

**CPRD flag for acceptable quality of data*

*^+^ data includes period outside practice registration with CPRD*

***no event date, episode start date after discharge, death prior to episode start, age>110*

1,015 robotic

40,622 laparoscopic

52,098 open

14,578 data from outside CPRD-registered period^+^
